# Homoepitaxial growth of isotopically enriched h^10^BN layers on h^11^BN crystals by high-temperature molecular beam epitaxy

**DOI:** 10.1038/s41699-025-00619-4

**Published:** 2025-11-19

**Authors:** Jonathan Bradford, Amy F. M. Collins, Tin S. Cheng, Jialiang Shen, James Kerfoot, Graham A. Rance, Jiahan Li, Christopher J. Mellor, Peter H. Beton, Guillaume Cassabois, Siyuan Dai, James H. Edgar, Sergei V. Novikov

**Affiliations:** 1 School of Physics and Astronomy, University of Nottingham, Nottingham, UK; 2 Materials Research and Education Center, Department of Mechanical Engineering, Auburn University, Auburn, AL USA; 3 Nanoscale and Microscale Research Centre (nmRC), University of Nottingham, Nottingham, UK; 4 School of Chemistry, University of Nottingham, Nottingham, UK; 5 Tim Taylor Department of Chemical Engineering, Kansas State University, Manhattan, KS USA; 6 Laboratoire Charles Coulomb UMR 5221 CNRS-Université de Montpellier, Montpellier, France; 7 Institut Universitaire de France, Paris, France

**Keywords:** Two-dimensional materials, Atomic force microscopy, Design, synthesis and processing

## Abstract

Isotope-enriched bulk hexagonal boron nitride (hBN) crystals have enhanced properties that improve the performance of nanophotonic and quantum technologies. Developing methods to deposit epitaxial layers on such crystals enables the exciting prospects of producing isotope-engineered hBN layers and heterostructures. Here, we demonstrate the homoepitaxial growth of hBN with phase-separated ^10^B and ^11^B isotopes by high-temperature molecular beam epitaxy (HT-MBE). Controlled nucleation, improved surface uniformity, and step-flow growth of an h^10^BN epilayer were achieved by etching the h^11^BN bulk crystals with molecular hydrogen. The alignment of the h^10^BN epilayer and host h^11^BN lattices was confirmed by lattice-resolved atomic force microscopy. Micro-Raman spectroscopy and scattering-type scanning near-field optical microscopy show that the bulk h^11^BN and h^10^BN epilayer have distinct phonon energies, with no intermixing of the van der Waals layers, thus enabling the different boron isotopes to be spatially separated in the heterostructure. This work demonstrates the potential of HT-MBE to produce isotopic heterostructures of hBN to advance future nanophotonic and quantum technologies.

## Introduction

Interest in hexagonal boron nitride (hBN) is rapidly increasing due to its unique electronic and optical properties, which have the potential to advance photonic and quantum technologies. As a wide bandgap semiconductor (~6 eV), it has potential as a deep-UV emitter^1–3^, and can host a variety of optically active defects that act as quantum emitters at room temperature^4–6^, some of which are magnetically addressable^7–9^. While the hexagonal polytype (AA’ stacked) is the most widely studied, sp^2^-hybridised BN can exist in metastable hexagonal (AA stacked), Bernal (AB stacked), or rhombohedral (ABC stacked) phases^10–12^. Such changes in the stacking order break the inversion symmetry present in AA’ hBN, altering its optical response (e.g., introducing nonlinearity)^10,11,13^, as well as separating the dipole moments of B-N pairs leading to piezoelectricity and ferroelectricity^14–17^.

Recently, isotope-enriched hBN crystals have proven beneficial for a variety of applications. For example, the high thermal neutron absorption cross-section of the ^10^B isotope and its transmutation to charge particles is useful for detecting neutrons^18,19^. The reduced masses of hBN change when enriched with ^10^B, ^11^B, ^14^N and ^15^N isotopes, which significantly impacts its phonon modes^20,21^, leads to improved thermal conductivity^22–24^, and enables hyperbolic phonon polariton dispersions engineered by isotope selection in van der Waals (vdW) structures^25–27^. Furthermore, the combination of ^10^B and ^15^N isotopes in h^10^B^15^N increases coherence times for the V_B_^-^ spin defect compared to hBN with natural isotope abundances^28,29^. Despite these many recent advances, isotopically enriched hBN remains available primarily in the form of millimetre-sized bulk crystals^20,30,31^. Therefore, practically all engineered vdW isotopic heterostructures have been produced by mechanically stacking flakes exfoliated from hBN crystals enriched with different boron isotopes, where the monoisotopic building blocks are multilayers and thin slabs^25^. Developing scalable methods to deposit isotopically-enriched hBN layers to produce heterostructures and superlattices with tailorable and contamination-free isotope interfaces is imperative for practical device applications.

Several approaches to wafer-scale growth of hBN thin films have been developed in recent years, including chemical vapour deposition (CVD) on metals^32–36^, metal-organic vapour-phase epitaxy (MOVPE)^11,12,37,38^ and high-temperature molecular beam epitaxy (HT-MBE)^39–43^. In material systems with established bulk crystal growth, homoepitaxy is used to grow thin films with higher quality than their bulk counterparts. The homoepitaxial growth of hBN has remained largely unexplored due to the limited availability of bulk hBN crystals, but there have been recent demonstrations of homoepitaxial growth of hBN on exfoliated hBN flakes by MOVPE^44^, and polycrystalline sp^2^-hybridised BN growth on polished pyrolytic BN (pBN) by CVD^45^. Of the currently available epitaxial growth methods, HT-MBE is distinctly advantageous for growing monoisotopic hBN, due to the readily available isotope-enriched elemental boron and nitrogen precursors.

Here we demonstrate the growth of vdW and lateral structures containing hBN layers with phase-separated ^10^B and ^11^B isotopes by homoepitaxial HT-MBE of h^10^BN layers on h^11^BN flakes exfoliated from isotopically-enriched bulk crystals^31^. Through a comprehensive analysis of the growth using atomic force microscopy (AFM), we show that the homoepitaxy of hBN proceeds by step-flow growth, requiring pre-treatment of exfoliated hBN by H_2_-etching to create monolayer step edges and promote nucleation. Using micro-Raman spectroscopy (μ-RS) and scattering-type scanning near-field optical microscopy (s-SNOM), we confirm the epitaxy of phase-separated h^10^BN layers, which opens prospects for practical isotope-engineered infrared nanophotonic devices and quantum technologies requiring spatial separation of nuclear spins.

## Results

### Molecular beam epitaxy of isotopically enriched BN

**Fig. 1:**
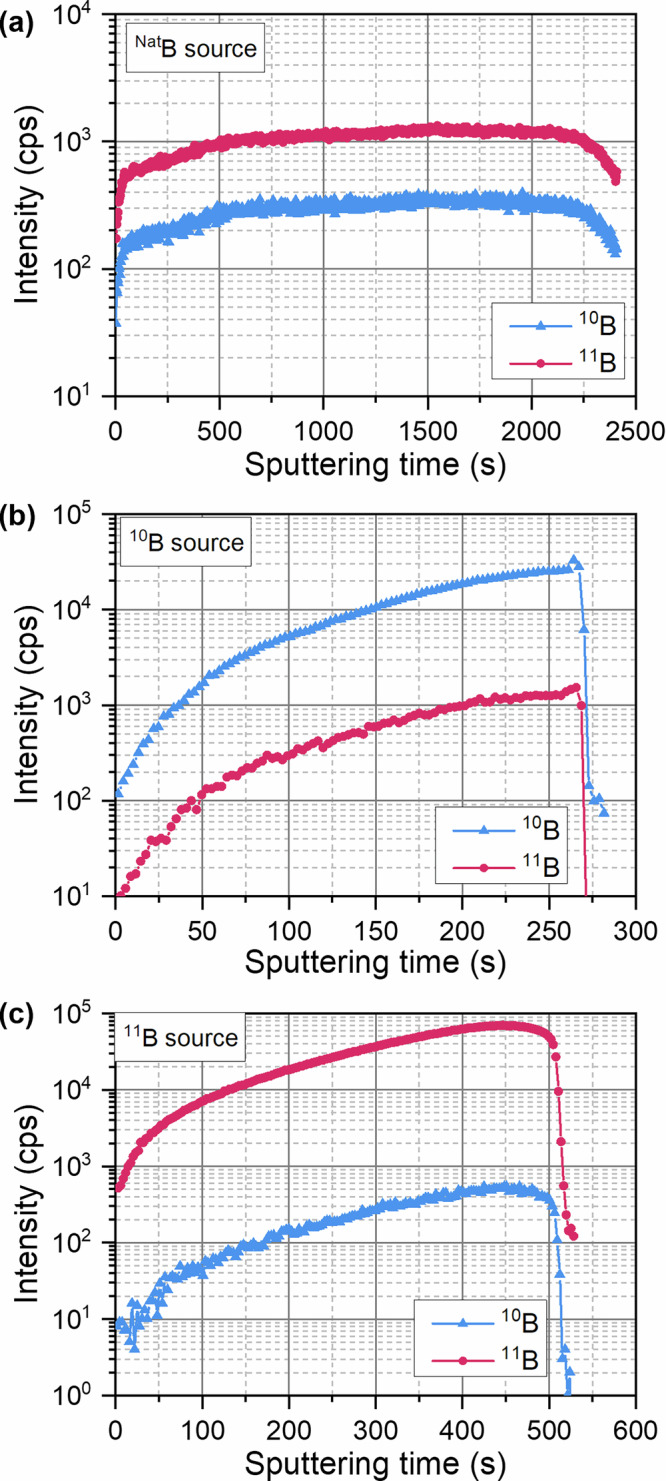
Secondary ion mass spectrometry. SIMS depth profiles of ^10^B and ^11^B isotopes in BN layers grown on sapphire with **a** ^Nat^B, **b** ^10^B, **c** ^11^B source material. Note that the different sputtering times required are due to different layer thicknesses for each sample.

To study ^10^B and ^11^B isotopically enriched epitaxy, we first separately grew BN layers on sapphire substrates by HT-MBE^1,2,41^ and explored the ratio of ^10^B to ^11^B using secondary ion mass spectrometry (SIMS). Figure 1 shows SIMS depth profiles of ^10^B and ^11^B of BN layers grown with a ^Nat^B source, comprising a naturally abundant boron isotope mixture (Fig. 1a), and isotopically enriched ^10^B and ^11^B sources (Fig. 1b and 1c, respectively). In the ^Nat^BN layers, the layers contained 78 ± 1% ^11^B and 22 ± 1% ^10^B, close to the naturally occurring abundance of ^11^B (80.1%) and ^10^B (19.9%). In the cases of the isotope-enriched B sources, we find isotope purities of 94.3 ± 0.8% and 99.3 ± 0.1% for layers grown from ^10^B and ^11^B sources, respectively, with no substantial variations as a function of the thickness of the layers. These values are broadly in line with the quoted isotope purity of the source material (see Experimental Details), noting that these values are subject to instrumental uncertainties due to the unavailability of a suitable calibration sample (see Supplementary Information for further discussion).

We demonstrated previously that during plasma-assisted MBE of AlGaN layers, there is unintentional doping of the layers with boron due to decomposition of the pBN parts of nitrogen RF plasma sources^46^. In order to check for any influence of such pBN decomposition on the isotopic purity of boron in our layers, we separately grew pure boron layers under similar MBE growth conditions in the absence of the nitrogen RF plasma source. SIMS studies demonstrate that the ratio of ^10^B and ^11^B isotopes in these layers remains practically the same with or without RF plasma source operation (see Fig. S1 and Table S1, Supplementary Information). Therefore, the influence of decomposition of pBN parts on the isotopic purity of our layers is negligible, and any isotopic impurities introduced from the RF plasma source can be considered in the doping regime and are below the sensitivity of the SIMS measurements. The ratio of ^10^B and ^11^B isotopes in the layers is controlled mainly by the isotopic purity of the initial boron source material.

### Homoepitaxial growth of hBN

**Fig. 2:**
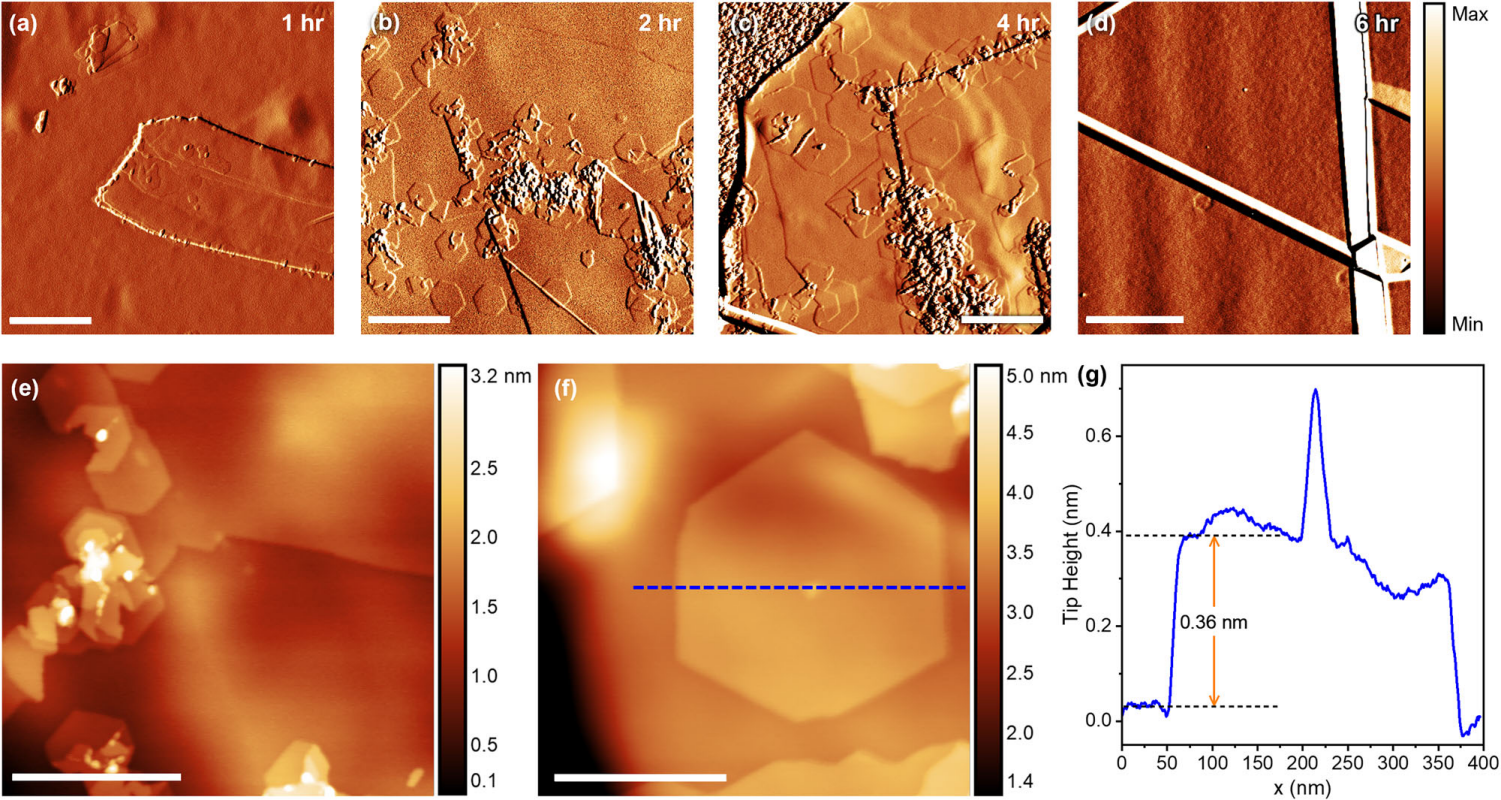
hBN homoepitaxy on pristine hBN flakes. Tapping mode AFM images of h^10^BN epilayers grown on exfoliated h^11^BN flakes for **a** 1 hr, **b** 2 hr, **c** 4 hr, **d** 6 hr. These images show the amplitude error signal, which improves step edge contrast against the flake morphology. **e** AFM topography of multilayer h^10^BN around defects. **f** AFM topography of an isolated h^10^BN island. The tip height profile from left to right along the dashed line is shown in (**g**). Scales bars are: **a**–**c** 500 nm, **d** 3 μm, **e** 400 nm, **f** 200 nm.

Next, we turn our attention to homoepitaxial growth of h^10^BN layers on h^11^BN crystals. Figure 2a–d shows atomic force microscopy (AFM) images of h^10^BN monolayers grown on exfoliated h^11^BN flakes mounted on sapphire as a function of growth time (1–6 h). The hBN flake morphology after growth is dominated by wrinkles formed during post-growth cooling (seen most clearly in Fig. 2d). These form due to the mismatch in thermal expansion coefficient between hBN and the sapphire substrate^47^. Because the structure of h^10^BN layers is nearly identical to h^11^BN, we search for the epilayers by identifying regions that are topographically atypical to exfoliated single crystal hBN flakes. Typically, the h^10^BN grows in small grains on the surface with mono- or few-layer thickness, and sometimes contains defects such as grain boundaries, or clusters of material on the surface, e.g., in Fig. 2e. Across numerous flakes and samples, there are occasionally hexagonal monolayer islands of h^10^BN nucleating on the surface, accompanied by a 3D defect in the centre of the island. An example of this is shown in Fig. 2f, with the tip height profile across the island shown in Fig. 2g, indicating monolayer thickness with 0.36 nm step height^43^. Precise identification of the object at the centre of the island is not possible by AFM; however, the height of the defect is 0.30 nm (less than the hBN layer thickness), suggesting it is some form of 3D cluster acting as a nucleation centre rather than the early growth of a second h^10^BN layer. After surveying multiple flakes on each sample, we frequently observed atomically flat surfaces of h^11^BN with a low density of structural defects, which limits the nucleation of the HT-MBE layer. Broadly, the growth of h^10^BN is both flake- and time-dependent, i.e., the nucleation, and hence coverage, of h^10^BN strongly depends on the initial flake topography. This is due to the nucleation being primarily at step edges on the surface, which are formed uncontrollably during hBN exfoliation. This effect is exemplified in Fig. 2d where, despite the longest growth time (6 h), the lack of steps or surface defects resulted in almost no h^10^BN growth.

**Fig. 3:**
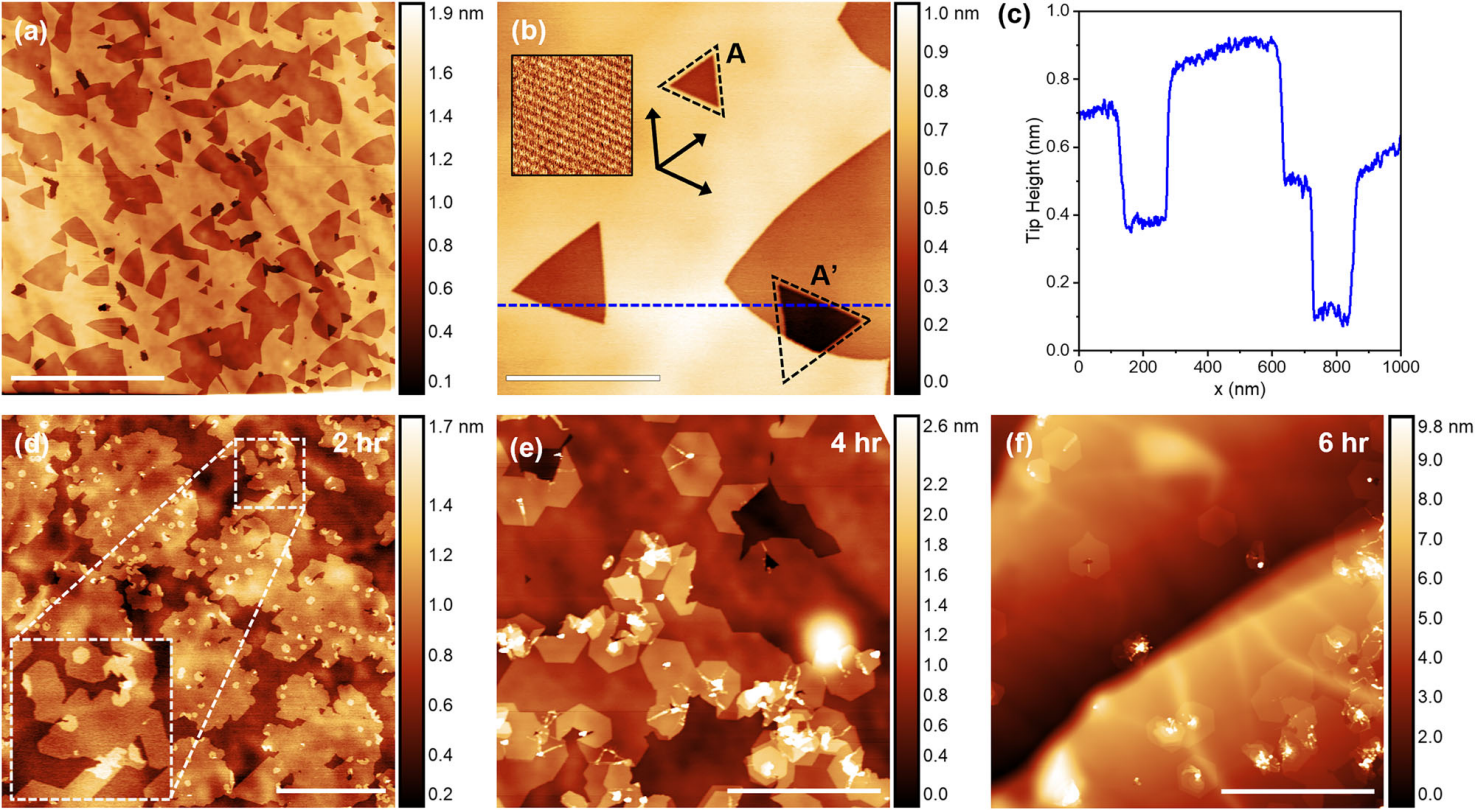
hBN homoepitaxy on etched hBN flakes. **a**, **b** Tapping mode AFM images showing the surface of a h^11^BN flake after H_2_-etching at 1000 °C for 8 h in flowing (0.15 slpm) 5% H_2_/Ar. The inset of (**b**) shows a lateral force microscopy (LFM) image of the hBN lattice on the top layer of the flake, and the black arrows indicate the h^11^BN lattice directions. The black dashed triangles reflect the 60° rotation between layers. **c** Tip height profile taken from left to right along the dashed line in (**b**). **d**–**f** AFM images of the surface of etched h^11^BN flakes after HT-MBE of h^10^BN for 2 hr, 4 hr and 6 hr, respectively. The inset in (**d**) shows a magnified view to highlight the faceted edges of the h^10^BN epilayer. Scale bars are **a** 4 μm, **b** 400 nm, **d**, **e** 1 μm, **f** 2 μm.

Next, we tested H_2_-etching the h^11^BN surfaces (see Experimental Methods) to increase the step edge density on the surface and thus the nucleation density. Representative AFM images of the h^11^BN surface after H_2_-etching are shown in Fig. 3a, b. The etching typically creates monolayer-deep pits in the surface with, occasionally, additional etching through subsequent hBN layers, shown in the tip height profile in Fig. 3c. The smallest pits have a sharp triangular shape with edges oriented along the zigzag lattice directions, as revealed by lattice-resolved contact-mode AFM imaging on the top layer. Previously, these have been proposed to be B-terminated edges when hBN monolayers on CuNi foils are etched in a H_2_/Ar atmosphere, and N-terminated edges when etched in H_2_ and N_2_, respectively^48^; however, this may differ in our work due to the use of bulk flakes and a non-catalytic substrate. Etching of the second layer also forms zigzag-oriented edges with the triangular pits rotated by 60° with respect to those in the first layer, reflecting the AA’ stacking in hBN (black dashed triangles in Fig. 3b). As the etching of the surface continues, the edges of the pits become rounded and do not follow any crystallographic orientation.

The evolution of non-faceted pits indicates that etching follows a non-equilibrium, dynamic process reminiscent of the growth of monolayer hBN islands by chemical vapour deposition (CVD) on metal surfaces. In the case of crystal growth under near-equilibrium conditions, the island shape will follow kinetic Wulff construction, resulting in triangular or truncated triangular islands depending on the boron and nitrogen precursor flux ratio^36,49,50^. At high precursor flux (non-equilibrium growth conditions), hBN islands grow with convex edges due to dynamic growth processes in which the attachment of adatoms to edge structures occurs at a comparable or higher rate than relaxation of the edge structure. We, therefore, propose that the H_2_-etching process seen here is also occurring under non-equilibrium conditions. Etch pits initially form a triangular shape with zigzag-oriented edges, as shown in Fig. 3b, and expand with convex edges due to the etch rate exceeding the relaxation of a zigzag edge. Importantly, the largest etch pits do not evolve into a hexagonal shape, nor form edges oriented along the armchair lattice directions. This supports the hypothesis of non-equilibrium etching conditions rather than an evolution towards armchair-terminated edges, which is typically the most favourable edge termination for hBN^51^.

Figure 3d–f show topographic AFM images of h^10^BN epilayers grown on the H_2_-etched h^11^BN flakes with increasing HT-MBE growth time. In contrast to growth on pristine h^11^BN flakes, HT-MBE of h^10^BN layers on the etched surface results in much more uniformly distributed growth, confirming that step edges play an important role in nucleation. Variations between flakes remain and reflect a flake-to-flake non-uniformity of the etched surfaces. For example, the nucleation density in Fig. 3d appears much higher than in Fig. 3f, although we cannot exclude the possibility that etched pits are completely over-grown after prolonged growth periods. In all instances, there are isolated hexagonal islands of monolayer thickness on the surface. As a function of time, the lateral size of isolated h^10^BN islands increases approximately linearly at a rate of ~130 ± 10 nm hr^−^^1^ (see Fig. S2, Supplementary Information).

**Fig. 4:**
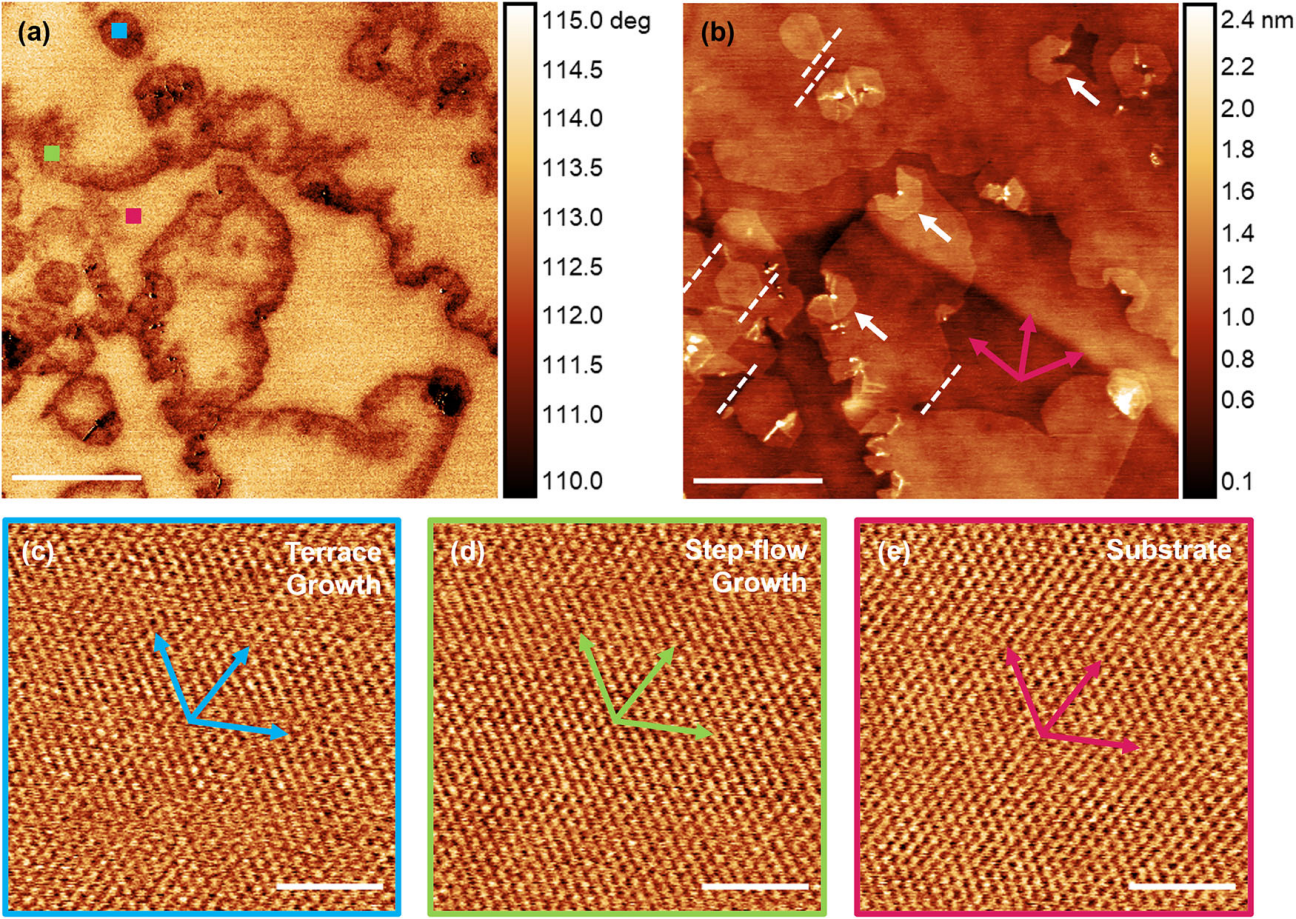
Step-flow growth mechanism. **a** Tapping mode AFM phase and **b** topography of h^10^BN grown on H_2_-etched h^11^BN for 4 h. **c**–**e** Lateral force microscopy (LFM) images of the hBN lattice acquired at the blue, green and pink points, respectively, indicated in (**a**). The lattice contrast has been enhanced by 2D-FFT filtering. Note that the LFM images are scanned at a 90° angle relative to the tapping mode image in (**a**). This corresponds to a scan direction perpendicular to the length of the cantilever, which maximises the lateral force signal. Scale bars are **a**, **b** 1 μm, **c**–**e** 2 nm.

Next, we examine the homoepitaxial growth modes and epitaxial relationship between the h^10^BN epilayer and h^11^BN substrate. Figures 4a and 4b shows tapping mode AFM phase contrast and topography, respectively, of a H_2_-etched surface after 4 h growth. Note that the boron isotope provides phase contrast to distinguish between ^10^B and ^11^B isotopes in hBN, which appear as smaller and larger phase shifts, respectively in Fig. 4a. This is probably due to the difference in the mechanical properties of the h^10^BN and h^11^BN layers^52^, although we note differences in the vdW interactions between the flake and grown layers may also contribute^53^. In addition to the growth of hexagonal h^10^BN islands nucleating at terrace defects, we also observe step-flow growth from the etched h^11^BN edges. This is consistent with previous observations of step-flow growth of hBN from monolayer step edges on HOPG^42,43^. h^10^BN islands also nucleate on the upper terrace of a h^11^BN step edge, as indicated by the white arrows in Fig. 4b. It is not clear whether these islands are h^10^BN bilayers formed where the step flow growth is pinned by a 3D defect (e.g., a boron aggregate), or monolayer growing on the upper terrace of a h^11^BN step edge.

Close inspection of facetted h^10^BN edges shows that the majority of the grown islands and step-flow growth fronts share a common orientation, marked by the white dashed lines in Fig. 4b, indicating a homoepitaxial growth relation of the h^10^BN layer to h^11^BN. To determine the atomic registry of the grown layer with the underlying h^11^BN we have examined the local orientation of the hBN lattices by lateral force microscopy (LFM). Figure 4c–e shows lattice-resolved LFM images of the hBN surface at three locations marked by the coloured boxes in Fig. 4a. These correspond to h^10^BN layers grown on a terrace (blue) and step edge (green), and the exposed h^11^BN flake surface (pink). These images reveal that the hBN lattices are all aligned and therefore the dominant orientation of the epilayer is 0° with respect to the underlying h^11^BN growth template. By comparing the lattice directions (pink arrows) with the orientation of the grown hBN facets, it can also be deduced that faceted edges of the grown h^10^BN are armchair-terminated. This indicates that the edge termination of the h^10^BN is not determined by the orientation of the nucleating step edge, and instead the edges evolve into their most energetically favourable configuration^51^, as is the case for HT-MBE hBN grown on HOPG^42,43^.

### Micro-Raman Spectroscopy (μ-RS) and Scattering-type Near-Field Optical Microscopy (s-SNOM) characterisations

**Fig. 5:**
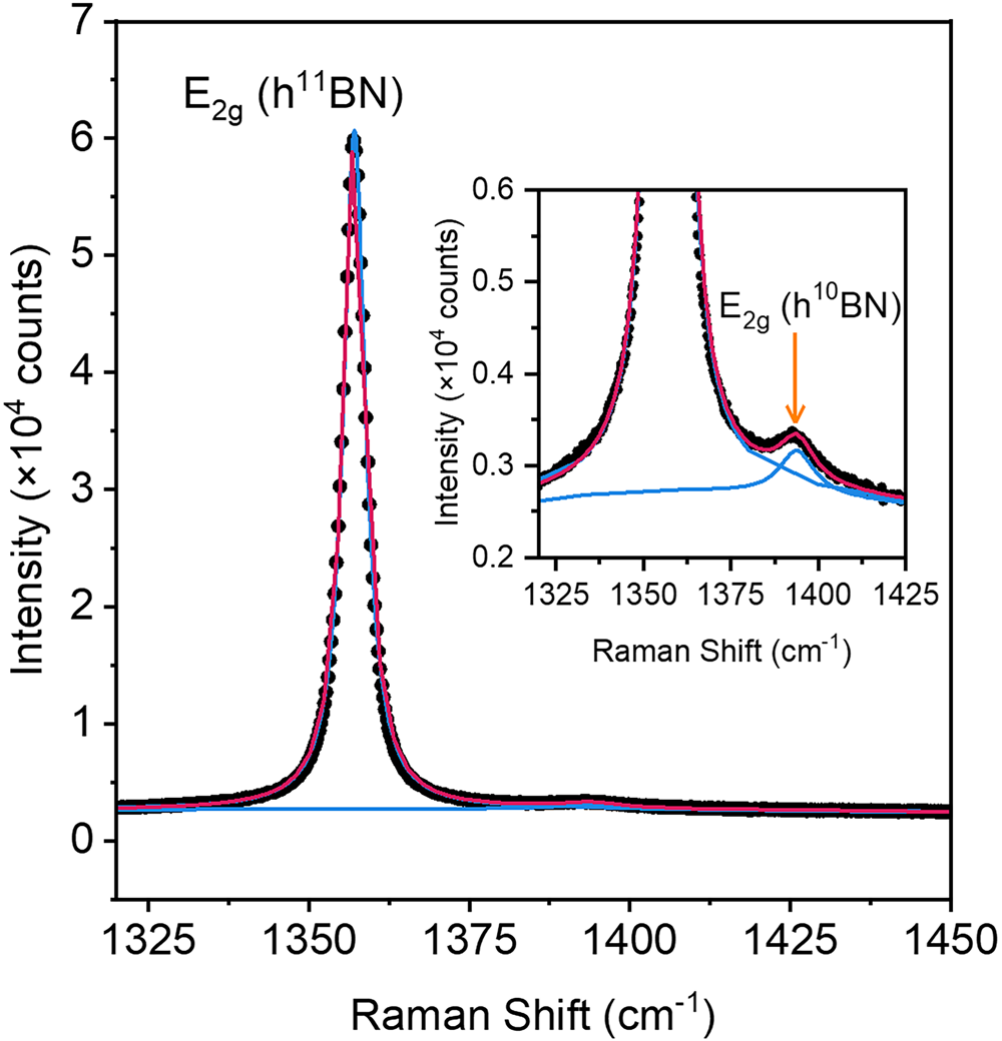
Micro-Raman spectroscopy. μ-RS analysis (λ = 532 nm) of epitaxial h^10^BN grown on a ~10 nm thick h^11^BN flake. Inset: magnified view of the spectrum showing the low-intensity h^10^BN signal.

The harmonic oscillator model informs us that the frequency of a given lattice vibration (ν) is inversely proportional to atomic mass (ν ~ √(*k*/*µ*)), where *k* is the force constant and *µ* the reduced mass). Consequently, vibrational spectroscopy analysis represents an ideal methodology to discriminate the different isotopes of B present in the MBE-grown flakes prepared. Belonging to the D_6h_ point group and comprising an irreproducible representation containing zone-centre phonon modes of E_1u_ + A_2u_ + 2E_2g_ + 2B_1g_ symmetry, the E_1u_ and E_2g_ modes of hBN, both involving in-plane atomic displacements, are infrared and Raman active, respectively, appearing in both cases at ~1357, 1366 and 1393 cm^−^^1^ for h^11^BN, h^Nat^BN and h^10^BN, respectively^20,21^. Figure. 5 shows μ-RS analysis of epitaxial h^10^BN on a ~10 nm thick h^11^BN flake after 4 hours growth. As expected, the spectrum is dominated by a peak at 1357.1 cm^−1^ corresponding to the h^11^BN flake; however, a second, weaker signal is also detected 1393.8 ± 0.4 cm^−1^ (uncertainty due to Lorentzian peak fitting), shown in the inset of Fig. 5, in good agreement with that expected for h^10^BN^21,30^. The h^10^BN:h^11^BN ratio of the peak areas is 0.024 ± 0.001, which would suggest just below a monolayer coverage of hBN within the laser spot, assuming the Raman scattering cross section is the same for the two isotopes, and minimal scattering losses from the lower layers. We note that very thin h^11^BN flakes are required to detect a signal from the h^10^BN epilayer due to the dominance of the h^11^BN signal. The FWHM of the h^10^BN peak is 12 ± 1 cm^−1^, which compares favourably with MOCVD-grown hBN layers (noting these contain natural isotope mixtures)^37,54^, but is broadened compared to isotopically purified bulk h^10^BN crystals (typically ~4 cm^−1^)^21,30^. We attribute the broadening to the small domain sizes (~200 nm) for the measured sample, which will result in grain boundaries and edges within the laser spot. A small degree of broadening is also expected due to the lower isotope purity of the ^10^B source compared to ^11^B source (see Experimental Details).

**Fig. 6:**
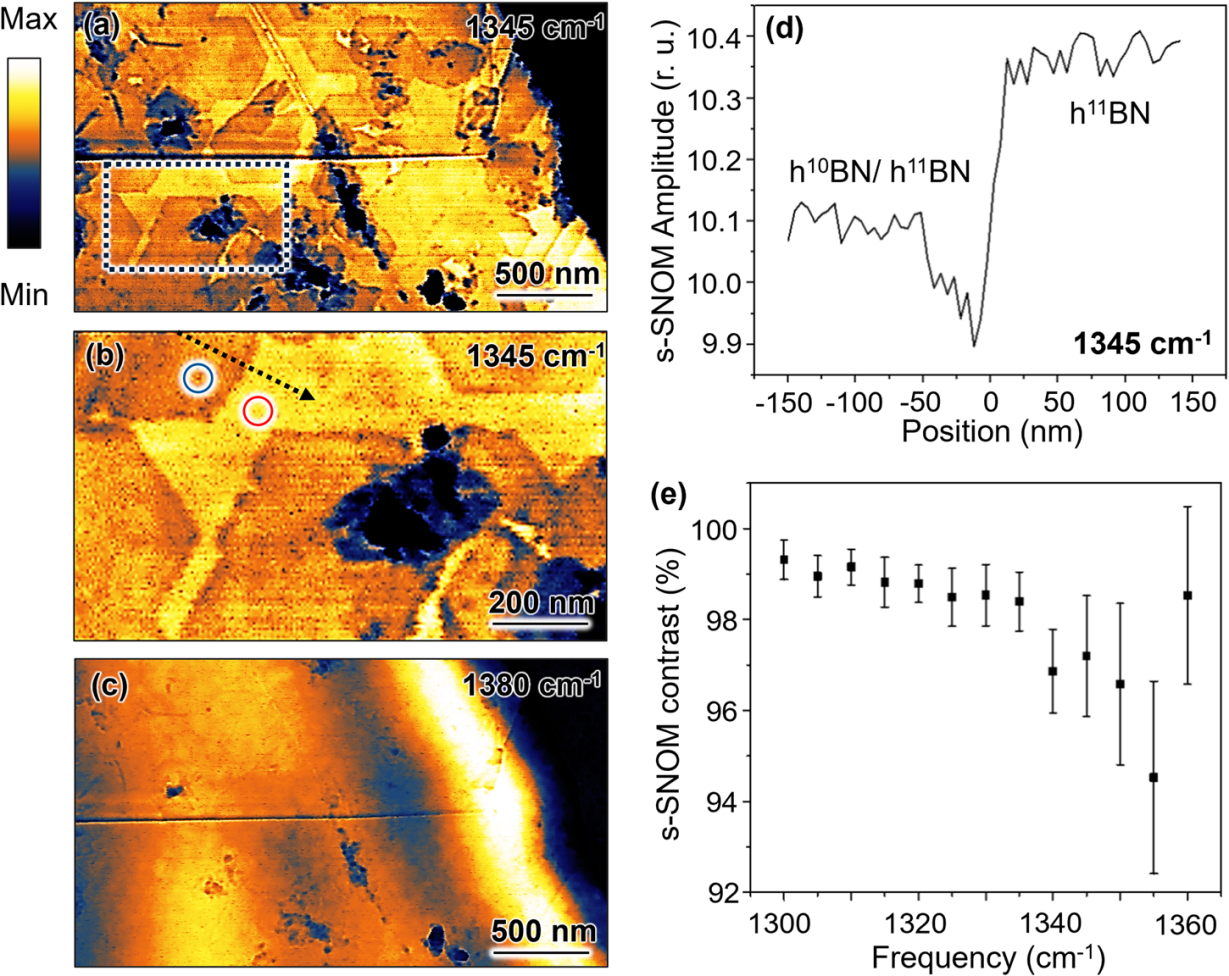
Spatial discrimination of h^10^BN and h^11^BN. s-SNOM amplitude images of epitaxial h^10^BN grown on h^11^BN at 1345 cm^−1^ (**a**, **b**) and 1380 cm^−1^ (**c**). **b** is the zoom-in image of the rectangular region in (**a**). **d** Line profile of the near-field amplitude extracted along the dashed arrow in (**b**). **e** s-SNOM contrast—s-SNOM amplitude ratio between the grown h^10^BN monolayer on h^11^BN (blue circle in (**b**)) and the pristine h^11^BN flake (red circle in (**b**))—at various frequencies. The squares represent mean values, with error bars indicating standard deviations.

Since μ-RS is limited by optical diffraction and the h^10^BN domains are smaller than the limiting spatial resolution, it was not possible to spatially discriminate h^10^BN and h^11^BN by μ-RS. Instead, the h^10^BN and h^11^BN layers were spatially resolved in the heterostructure using s-SNOM at frequencies near the transverse optical (TO) phonon of the E_1u_ mode in h^11^BN (Fig. 6). Since the TO resonances vary with different isotopes, the epitaxially grown h^10^BN monolayers show contrast in the s-SNOM amplitude images. At a representative frequency of 1345 cm^−1^, the hexagonally shaped h^10^BN monolayers exhibit lower contrast in the s-SNOM image (Fig. 6a) and its zoomed-in view (Fig. 6b). The extracted line profile (Fig. 6d) along the black dashed arrow in Fig. 6b reveals a ~3% reduction in s-SNOM amplitude for grown h^10^BN monolayers on h^11^BN compared to a pristine h^10^BN flake. This s-SNOM contrast of the grown h^10^BN is evident at frequencies (Fig. 6e) below the h^11^BN TO phonon (1357 cm^−1^) since these imaging frequencies are closer to the h^11^BN resonance rather than the h^10^BN TO resonance at 1393 cm^−1^. A systematic s-SNOM imaging study on the epitaxially grown h^10^BN/h^11^BN heterostructure shows the s-SNOM contrast—s-SNOM amplitude ratio between the grown monolayer h^10^BN/h^11^BN and pristine h^11^BN—varies with frequency. As the frequency increases, the contrast decreases, reaching a minimum at the h^11^BN TO phonon (1357 cm^−1^) before rising as it approaches the h^10^BN TO phonon (1393 cm^−1^). At another representative frequency of 1380 cm^−1^ (Fig. 6c), the s-SNOM contrast between the grown monolayer h^10^BN/h^11^BN and pristine h^11^BN becomes negligible, and phonon polariton standing wave fringes appear near the sample edge.

## Discussion

In this work, we have elucidated the step-flow hBN homoepitaxial growth mechanism by examining the initial stages of growth in the presence and absence of steps on the hBN surface. These findings are in line with previous demonstrations of step-flow growth of hBN on graphene^42,43^ and vice versa^55^. We anticipate that our approach of H_2_-etching to improve nucleation of the hBN epilayer will be relevant for HT-MBE and MOVPE alike. Recent studies of hBN homoepitaxy by MOVPE showed growth of multilayer hBN primarily at the edges of exfoliated hBN flake edges^44^. Furthermore, nucleation from zigzag-oriented flake edges forced the growth of non-centrosymmetric BN layers with Bernal stacking (bBN). Thus, the formation of zigzag step edges by H_2_-etching, as demonstrated in this work, may provide a route to deterministic growth of bBN crystals. We have attempted to identify the layer stacking between the h^10^BN epilayer and h^11^BN flakes in this work by photoluminescence (PL) and second-harmonic generation (SHG)^10^; however, these measurements were not successful, likely due to the predominantly monolayer thickness of the epilayer. At present, the main limitation of HT-MBE growth is the slow growth rates on van der Waals surfaces, which limit the epilayer thickness to a few layers. Achieving higher growth rates by increasing the boron flux or reducing the substrate temperature (to reduce desorption of adatoms from the surface) comes at the expense of the structural quality of the layers, which contain more grain boundaries and 3D aggregates^43^. Faster growth rates can be achieved on sapphire substrates (~4 nm/h) under identical growth conditions, but also with reduced crystalline quality, producing polycrystalline layers with weak optical response^1,41^.

Developing HT-MBE growth of isotopically enriched hBN, as demonstrated in this work, expands the possibility of growing hBN layers with mixed or single isotopes at scales compatible with device fabrication. The use of elemental B and N sources in HT-MBE makes it a uniquely suitable and versatile tool to grow on hBN thin films and heterostructures with controlled isotope compositions. By contrast, vapour-based growth methods (e.g., CVD and MOCVD), which are the most widely used methods for growing hBN thin films, require specialised precursors that are not readily available in an isotopically purified form. Recent demonstrations of ultrafast heat transfer across solid-solid interfaces via phonon-polariton coupling^56^, and improved thermal conductivity in isotopically pure hBN (including at the monolayer limit)^22–24^, make this system highly appealing for thermal management in future 2D electronic systems^57^. Additionally, our results showing the growth of phase-separated h^10^BN and h^11^BN heterostructures are promising for emerging nanophotonic technologies that utilise hyperbolic phonon polaritons in isotope-engineered van der Waals and lateral heterostructure configurations. Overall, we expect this work to stimulate advances in isotope-controlled hBN synthesis using wafer-scale growth processes.

## Methods

### Growth of isotopically enriched Bulk h^11^BN crystals

The bulk monoisotope h^11^BN crystalline flakes were grown using an iron-chromium flux and elemental ^11^B-enriched boron (99.41 at%) as described in ref. ^18^. After evacuating and purging the furnace, powders of the source materials were placed in an alumina crucible, then heated to 1550 °C under flows of 700 sccm of N_2_ (natural isotope abundance, 99.6% ^14^N and 0.4% ^15^N) and 30 sccm of forming gas (95% Ar and 5% H_2_), at 820 Torr. The mixture was held at this temperature for 24 hours, to ensure the solution composition was well-mixed and uniform. To grow crystals, the solution was slowly (4 °C h^−^^1^) cooled to 1450 °C, then rapidly cooled (200 °C h^−1^) to room temperature. Freestanding h^11^BN crystals (10 to 20 µm thick) were produced by exfoliating them from the metal ingot using thermal release tape.

### High-temperature molecular beam epitaxy

Epitaxial h^10^BN layers were grown by HT-MBE using a custom-designed Veeco GENxplor system capable of achieving substrate temperatures up to 1850 °C (measured by thermocouple)^1,41^. An active nitrogen flux was provided by a Veeco RF plasma source operated at a fixed RF power of 550 W and N_2_ (natural isotope abundance, 99.6% ^14^N and 0.4% ^15^N) gas flow rate of 2 sccm. The boron flux was produced by a Veeco high-temperature Knudsen effusion cell operating at 1875 °C. The cell was loaded with isotopically enriched ^10^B (purity >96%) or ^11^B (purity >98%). All samples studied in this work were grown at a substrate heater temperature of 1390 °C, with the growth time ranging from 1–6 h to achieve variable coverage.

Isotopically enriched h^11^BN flakes were mechanically exfoliated onto single-side polished sapphire wafers and soaked in toluene for 4 hours to remove tape residues. Prior to HT-MBE, samples were cleaned by annealing for 8 hours in a flowing H_2_/Ar (5%, 0.15 slpm) atmosphere at 600 °C to preserve the exfoliated surface, or at 1000 °C for H_2_-etching.

### Atomic force microscopy

AFM measurements were conducted using an Asylum Research Cypher S microscope in ambient conditions. Tapping mode and lateral force microscopy images were acquired using Si cantilevers with a nominal spring constant of 2 Nm^−1^ (SCOUT 70 RAl, NuNano).

### Secondary ion mass spectrometry

The chemical concentrations of B and N were studied as a function of depth using SIMS in two commercial systems, namely a Cameca IMS-3F and a Cameca IMS-7F. The ^10^B and ^11^B isotope analysis was carried out using O_2_^+^ primary ion bombardment and positive secondary ion detection to optimise sensitivity to boron. The data were not calibrated due to a lack of suitable reference calibration standards.

### Scattering-type scanning near-field optical microscopy

The nano-imaging of epitaxially grown h^10^BN/h^11^BN was conducted using the scattering-type scanning near-field optical microscope (s-SNOM) from Attocube. The s-SNOM is based on a tapping-mode AFM. In the experiments, the PtIr-coated AFM tip with a radius of ~10 nm (Arrow-NCPt, NanoWorld AG, Switzerland) was illuminated by monochromatic Mid-IR quantum cascade lasers (QCLs, www.daylightsolutions.com) with frequency spanning 845 to 1800 cm^−1^. The s-SNOM images were recorded by a pseudoheterodyne interferometric detection module with a tapping frequency of 245–280 kHz and tapping amplitude of ~70 nm. The detected optical signal was demodulated at the fourth harmonics of the tapping frequency in order to obtain the pure near-field signal.

### Micro Raman spectroscopy

Raman spectroscopy measurements were acquired with a HORIBA LabRAM Evolution Raman spectrometer using 532 nm laser excitation focused through a 100× objective (NA 0.9) and a 100 μm confocal pinhole. A rotatable diffraction grating with 1800 lines mm^−1^ and a Synapse CCD were used for detection, resulting in a pixel separation of <0.3 cm^−1^ (as measured at the low-energy cut-off). Raman spectra were calibrated using a polystyrene reference mounted on the instrument objective turret (SP-RCO).

## Supplementary information

Supplementary information

## Data Availability

The data that support the findings of this study are openly available at the following URL/DOI: 10.17639/nott.7593.
