# Supplementary material for: Homoepitaxial growth of isotopically enriched h10BN layers on h11BN crystals by high-temperature molecular beam epitaxy
Source: NPJ 2D Mater Appl. 2025 Nov 19;9(1):98. doi: 10.1038/s41699-025-00619-4 (PMC12629975; doi:10.1038/s41699-025-00619-4)
Supplement: Supplementary file 1 — Supplementary information [file 41699_2025_619_MOESM1_ESM.pdf]

Supplementary Information

Homoepitaxial Growth of Isotopically Enriched $h^{10}BN$ Layers on $h^{11}BN$ Crystals by High-Temperature Molecular Beam Epitaxy

Jonathan Bradford[1,*], Amy F. M. Collins[1], Tin S. Cheng[1], Jialiang Shen[2], James Kerfoot[3], Graham A. Rance[3,4], Jiahan Li[5], Christopher J. Mellor[1], Peter H. Beton[1], Guillaume Cassabois[6,7], Siyuan Dai[2], James H. Edgar[5], Sergei V. Novikov[1,*]

[1] School of Physics and Astronomy, University of Nottingham, Nottingham NG7 2RD, United Kingdom

[2] Materials Research and Education Center, Department of Mechanical Engineering, Auburn University, Auburn, AL 36849, USA

[3] Nanoscale and Microscale Research Centre (nmRC), University of Nottingham, Nottingham, NG7 2RD, United Kingdom

[4] School of Chemistry, University of Nottingham, Nottingham, NG7 2RD, United Kingdom

[5] Tim Taylor Department of Chemical Engineering, Kansas State University, Manhattan, KS 66506, USA

[6] Laboratoire Charles Coulomb UMR 5221 CNRS-Université de Montpellier, 34095 Montpellier, France

[7] Institut Universitaire de France, 75231 Paris, France

[*] Email: jonathan.bradford@nottingham.ac.uk; sergei.novikov@nottingham.ac.uk

Additional Secondary Ion Mass Spectrometry (SIMS) Data

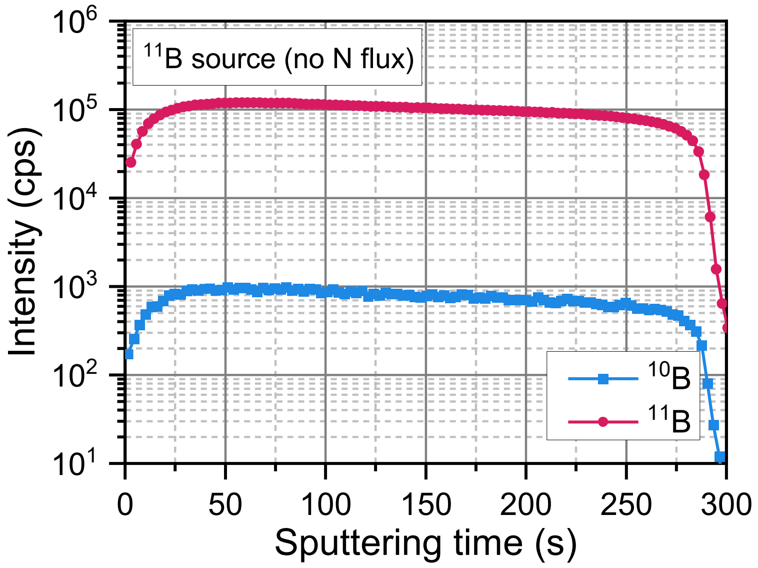

Figure S1. SIMS depth profile of a ^{11}B layer deposited on sapphire, with no nitrogen flux.

Table S1. Quantification of B isotope composition of BN and B layers grown from B sources with different isotope compositions.

Source	^{10}B Concentration (%)	^{11}B Concentration (%)
**^{10}B**	94.3 ± 0.8	5.7 ± 0.8
**^{Nat}B**	22 ±1	78 ±1
**^{11}B**	0.7 ± 0.1	99.3 ± 0.1
**^{11}B (no N flux)**	0.8 ± 0.2	99.2 ± 0.2

Since suitable reference standards were not available for the measurement, we can assess the accuracy based on the quantification of the ^{10}B and ^{11}B isotopes in the ^{Nat}B source. These should reflect the natural isotope abundance of ^{11}B (80.1 %) and ^{10}B (19.9 %). Our measurements underestimate this, with a measured abundance of 78±1 % and 22±1 % for ^{11}B and ^{10}B, respectively. This suggests that relative errors of up to 13.5% in the ^{11}B:^{10}B ratio could be present in our measurements. This could be improved by accurately determining the instrument bias correction factor,[1,2] however we anticipate that charging effects may also be factor. Nonetheless, the uncalibrated results clearly indicate the isotopic purity of the layers grown with enriched B sources.

Distribution of Lateral Size of $h^{10}BN$ Islands

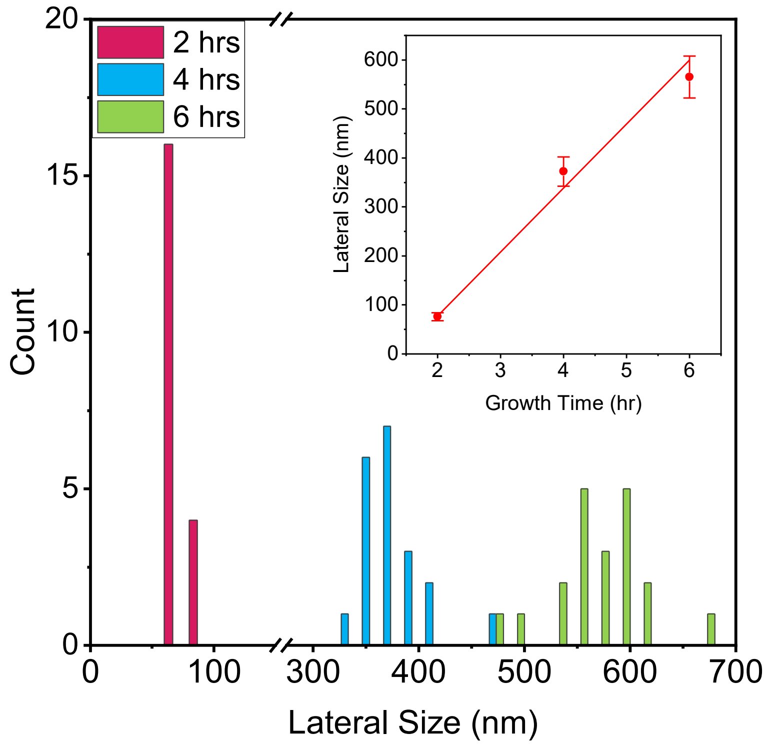

Figure S2. Distribution of the lateral size of isolated $h^{10}BN$ islands as a function of growth time on etched $h^{11}BN$ surfaces.

References

1. Christie, W. H., Eby, R. E., Warmack, R. J. & Landau, L. Determination of Boron and Lithium in Nuclear Materials by Secondary Ion Mass Spectrometry. *Anal Chem* **53**, 13–17 (1981).

2. Karki, V., Singh, M., Bhushan, K. S., Rao, R. M. & Jaison, P. G. Development of methodology for determining the isotopic composition of boron in powder and solid nuclear materials using secondary ion mass spectrometer. *Int J Mass Spectrom* **460**, 116475 (2021).
